# Relationship Between Acromial Anatomy and Rotator Cuff Tears in Saudi Arabian Population

**DOI:** 10.7759/cureus.8304

**Published:** 2020-05-26

**Authors:** Abdulraheem A Almokhtar, Ahmed S Qanat, Albarra Mulla, Ziyad Alqurashi, Ahmed Aljeraisi, Adel H Hegaze

**Affiliations:** 1 Orthopedics, Faculty of Medicine, King Abdulaziz University, Jeddah, SAU

**Keywords:** acromial anatomy, rotator cuff tears

## Abstract

Objectives

We investigated the relationship between acromial shape, classified as Type I-IV by magnetic resonance imaging, and the occurrence and characteristics of rotator cuff tears (RCTs).

Methods

This retrospective cohort study included 89 patients aged 25 - 60 years who underwent RCT surgeries in the Orthopedic Department at King Abdulaziz University Hospital (KAUH) from January 2014 to April 2019. We collected imaging findings from the KAUH record system, which were then entered into a Google form (Google, Inc., Mountain View, CA) and exported to Microsoft Excel 2016 (Microsoft^®^ Corp., Redmond, WA). Correlations between variables were assessed using Chi-squared tests.

Results

The supraspinatus muscle in both men and women was most commonly affected by RCTs, accounting for 73.6% of all tears. Subscapularis was the next most commonly injured muscle of the rotator cuff (15.1%), followed by the infraspinatus muscle (11.3%). The majority of supraspinatus, infraspinatus, and subscapularis tears (69.2%, 66.7%, and 56.3%, respectively) were associated with flat acromia. In all cases, tears in association with flat acromia were more prevalent among women (supraspinatus: 51.3% in women, 17.9% in men, p = 0.030; infraspinatus: 50% in women, 16.7% in men, p = 0.292; subscapularis: 43.8% in women, 12.5% in men, p = 0.054).

Conclusions

No correlation exists between acromial shape and sex, regardless of the specific muscle injured. However, supraspinatus injury, acromial shape, and sex are significantly related; right-side partial tear injuries occur more frequently among women aged ≥ 50 years with flat acromia than other RCTs.

## Introduction

Rotator cuff disease and impingement syndrome are among the leading causes of shoulder pain and disability. The rotator cuff comprises a group of four muscles (the supraspinatus, infraspinatus, subscapularis, and teres minor) that maintain the stability and strength of the shoulder joint. Of these four muscles, the most commonly injured is the supraspinatus tendon [1].

The pathogenesis of a rotator cuff tear (RCT) is multifactorial; however, there are numerous risk factors for the injury, which are classified as either intrinsic or extrinsic. Intrinsic factors include degenerative changes, hypovascularity, and collagen fiber abnormalities. Extrinsic factors include subacromial impingement, stretch overload, the shape of the acromion, and the formation of acromial spurs [2].

The acromial shape can be divided into four types, described using the Bigliani classification: Type I describes a flat shape, Type II is curved, and Type III is hooked [3]. Two years after the original classification was developed, Gagey et al. added Type IV to the classification system, which describes a convex inferior surface [4].

Many studies have investigated the relationship between acromial shape and the incidence of RCT [2-4]. Some of these studies also analyzed the relationship between the presence of an acromion spur and the occurrence of impingement syndrome, which was first described by Neer [5]. In 1972, Neer claimed that acromial shape, specifically, the shape of anterior and inferior parts, was responsible for impingement of the rotator cuff and, therefore, advocated for the utility of anterior acromioplasty to enlarge the subacromial space and decompress the rotator cuff. Numerous studies have supported Neer’s theory of extrinsic impingement leading to cuff disease [2-3, 6]. A recent study involving 104 cases reported the predominant acromial shape to be curved (Type II) and the majority of patients to be female, suggesting this scenario to be the most common cause of shoulder impingement [7]. A study conducted in 2012 suggested that acromial spurs were risk factors for full-thickness RCTs [8]. 

Imaging techniques, such as magnetic resonance imaging (MRI), are routinely used to evaluate the relationship between acromial shape and RCTs and impingement [9-12]. Some researchers have compared the results of radiographic and MRI examinations for the determination of acromial shape, while others have used magnetic resonance arthrography and ultrasound (US) to diagnose RCTs [8-14]. In the clinic, MRI is the most widely used tool for evaluating the presence and size of RCTs in order to assess the suitability for conservative treatment or requirement for surgery [9-12].

To date, there have been no studies on the association between acromial shape and RCTs in a Saudi Arabian population. The present study aimed to assess this association among adult patients who were treated at King Abdulaziz University Hospital (KAUH) in Jeddah, Saudi Arabia.

## Materials and methods

Ethical statement

The Biomedical Ethics Research Committee approved the study and all its protocols of KAUH (Approval number: 470-19).

Study design and population

This retrospective cohort study recruited patients with full or partial RCTs, aged 25 - 60 years, who were admitted to the Orthopedic Department of KAUH in Jeddah in the western region of Saudi Arabia for surgical repair of the rotator cuffs between January 2014 and April 2019. We assessed the presence of RCTs using a retrospective review of MRI results from KAUH records. The exclusion criteria were as follows: (1) post-traumatic shoulder pain (due to fracture or otherwise); (2) any other shoulder surgery; (3) osteoarthritis of the shoulder joint; (4) inflammatory arthritis; (5) congenital deformity; (6) unstable or frozen shoulder; (7) septic shoulder; (8) bone tumors; and (9) calcified tendinitis. Data were collected from electronic records of KAUH and then entered into a Google form (Google, Inc., Mountain View, CA, USA) and exported to Microsoft Excel 2016 (Microsoft® Corp., Redmond, WA, USA). We collected demographic data (sex, age, medical record number); no names were obtained to preserve patient confidentiality. We recorded the side of injury (right or left), acromial shape (flat, curved, hooked, or convex inferior surface), type of RCTs (partial or complete), and the muscle injured (supraspinatus, infraspinatus, subscapularis, and teres minor).

Statistical analysis

Categorical data are expressed as frequencies (percentage), and continuous data are expressed as means and standard deviations. The Chi-square test was used to calculate the correlations between variables. Statistical analysis was performed using the IBM Statistical Package for Social Sciences (SPSS), version 21.0 (IBM SPSS Statistics, Armonk, NY, USA).

## Results

**Figure 1:**
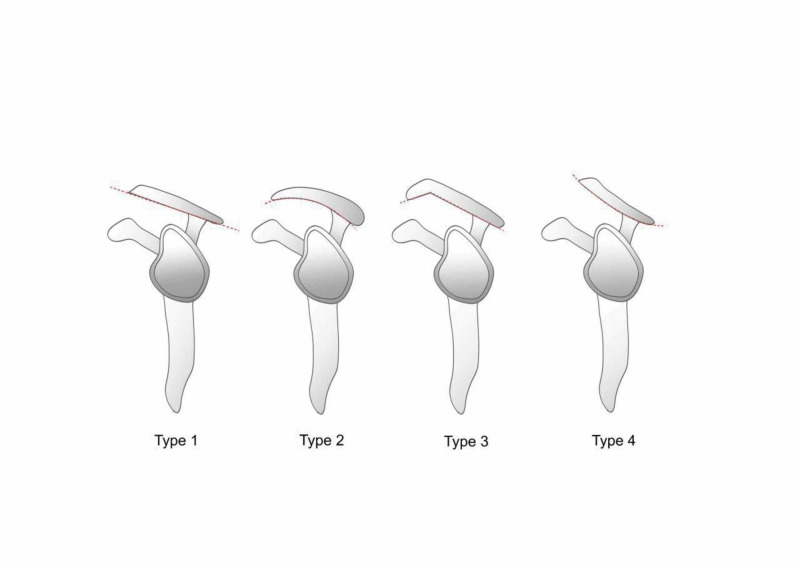
Types of acromial shapes

**Figure 2:**
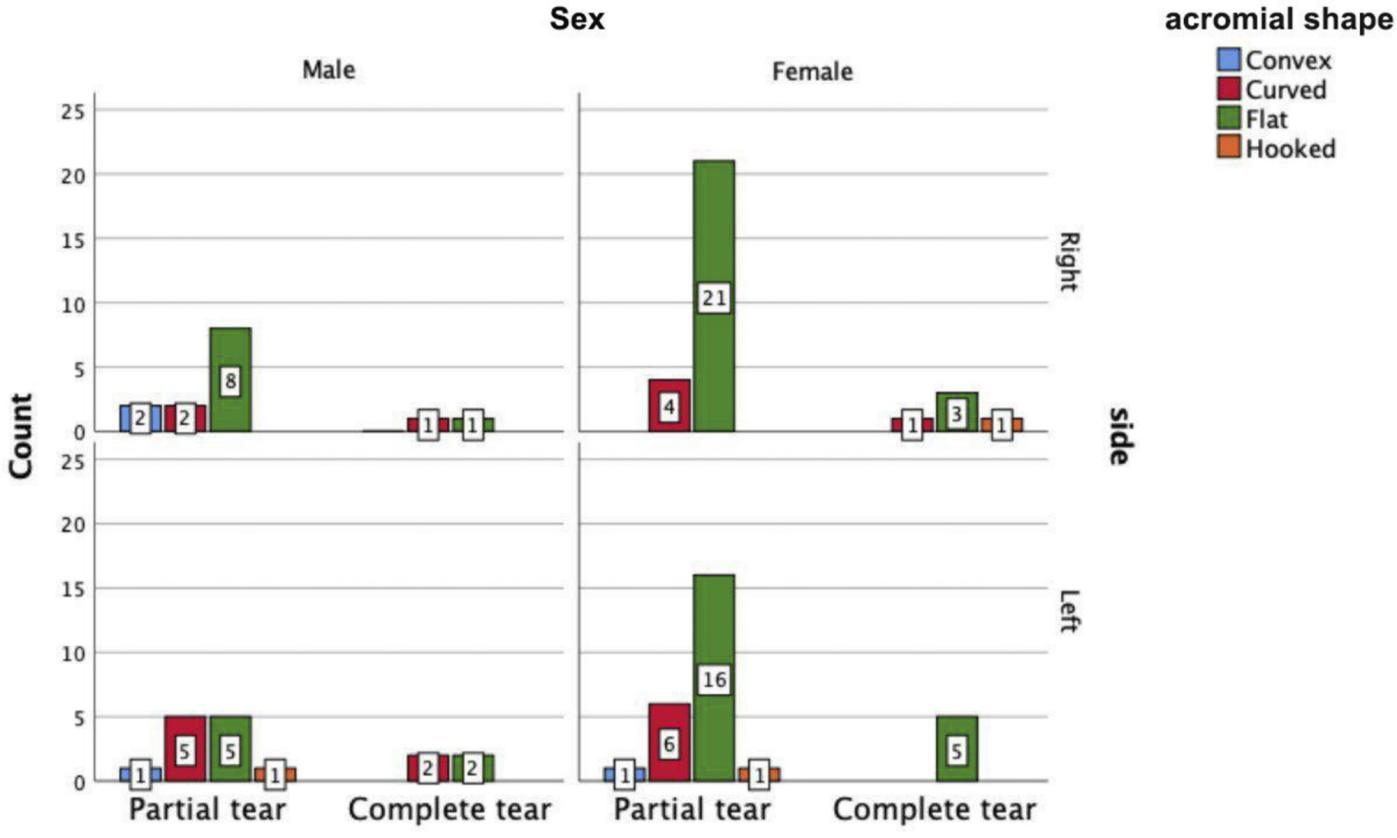
The relation between acromial shape and type of RCT in regard to the sex and side

In total, 954 patients underwent MRI of the shoulders due to pain. Of these, we recruited 89 patients (30 men and 59 women) with RCTs for this study. Some had more than one muscle injury. The mean age was 51.84 (standard deviation (SD): 8.312) years. We identified all four types of acromia based on MRI results (Figure 1). Injuries to the left shoulder were more common than those to the right shoulder among male patients, while the right side was more commonly affected in women (Figure 2).

**Figure 3:**
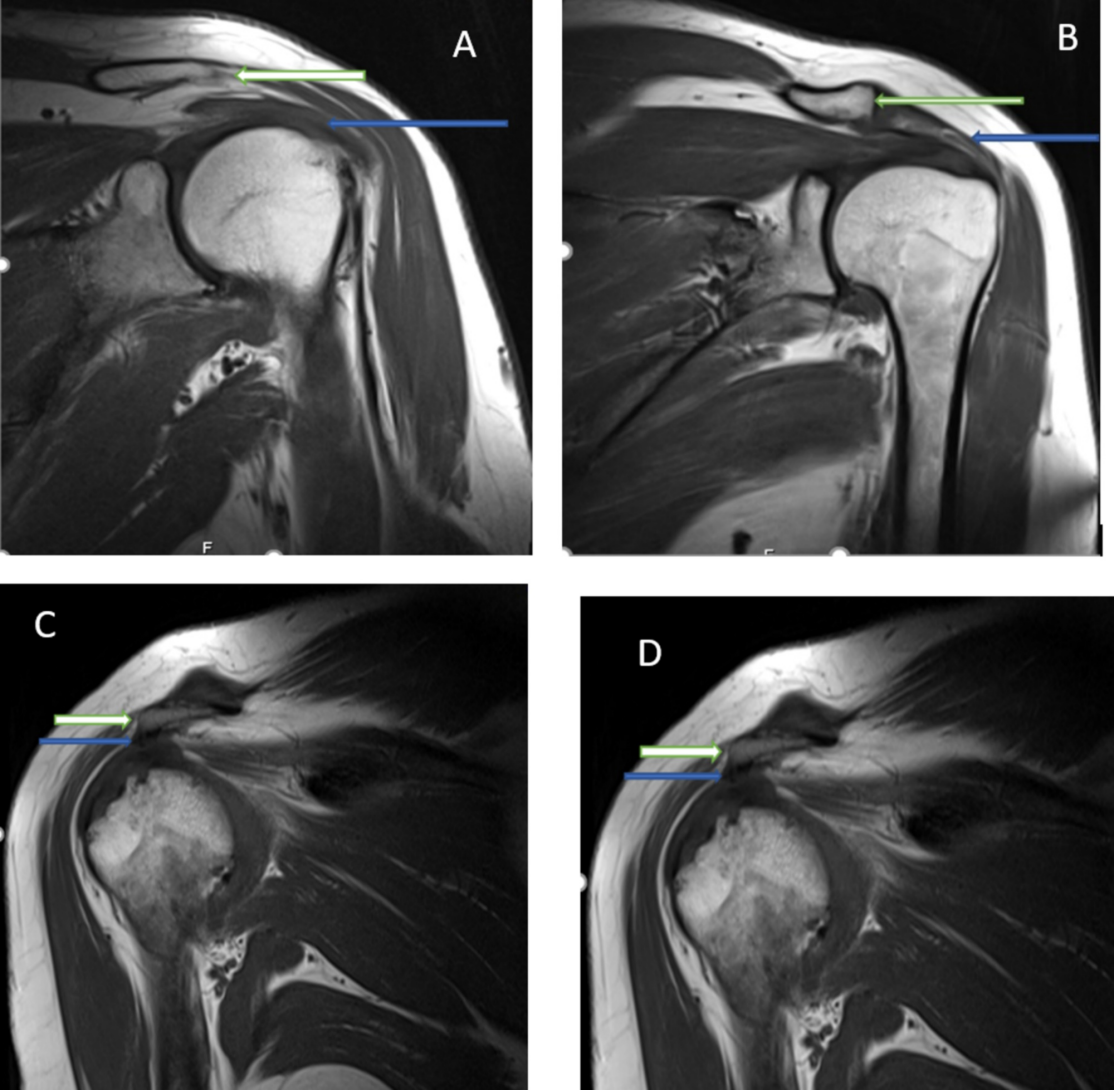
Types of acromial shapes associated with muscle injuries A: Type I acromion (flat): green arrow; supraspinatus complete tear: blue arrow B: Type II acromion (curved): green arrow, partial supraspinatus tear: blue arrow C: Type IV acromion (convex): green arrow, partial supraspinatus tear: blue arrow D: Type III acromion (hooked): green arrow, supraspinatus complete tear: blue arrow

The flat was the most common shape to be associated with RCTs in both male (53.3%) and female (76.3%) patients (p = 0.104), while the hooked shape was the least prevalent in men (3.3%) and the convex shape was least prevalent in women (1.7%) (Figures 2, 3A).

The most common muscle to be injured was the supraspinatus (73.6%) among both male (33.3%) and female (66.7%) patients (Table 1). The majority of these injuries occurred without other muscles being affected (p = 0.030) (Table 2). Figure 3A-D presents MRI images of the observed muscle injuries. Conversely, subscapularis and infraspinatus muscle injuries were more commonly found in combination with other RCTs than alone (p = 0.292 and 0.054, respectively).

**Table 1:** Frequency of Rotator Cuff Injury RTCs: rotator cuff tears

		Frequency	Percent	Nature of injury alone vs. in combination with other RCTs (%/%)
Muscles	Infraspinatus muscle	12	11.3	25 vs. 75
	Subscapularis muscle	16	15.1	43.75 vs. 56.25
	Supraspinatus muscle	78	73.6	80.77 vs. 19.23
	Total	106	100.0	

**Table 2:** Distribution of Rotator Cuff Muscle Injuries in Relation to Acromial Shape and Sex

				Sex		Total
				Male	Female	
Supraspinatus muscle	Acromial shape	Convex	Count	3	0	3
			% of total	3.8%	0.0%	3.8%
		Curved	Count	8	10	18
			% of total	10.3%	12.8%	23.1%
		Flat	Count	14	40	54
			% of total	17.9%	51.3%	69.2%
		Hooked	Count	1	2	3
			% of total	1.3%	2.6%	3.8%
	Total		Count	26	52	78
			% of total	33.3%	66.7%	100.0%
	P-value					.030
Infraspinatus muscle	Acromial shape	Convex	Count	0	1	1
			% of total	0.0%	8.3%	8.3%
		Curved	Count	2	1	3
			% of total	16.7%	8.3%	25.0%
		Flat	Count	2	6	8
			% of total	16.7%	50.0%	66.7%
	Total		Count	4	8	12
			% of total	33.3%	66.7%	100.0%
	P-value					.292
Subscapularis muscle	Acromial shape	Convex	Count	1	0	1
			% of total	6.3%	0.0%	6.3%
		Curved	Count	4	1	5
			% of total	25.0%	6.3%	31.3%
		Flat	Count	2	7	9
			% of total	12.5%	43.8%	56.3%
		Hooked	Count	1	0	1
			% of total	6.3%	0.0%	6.3%
	Total		Count	8	8	16
			% of total	50.0%	50.0%	100.0%
	P-value					.054

Supraspinatus, infraspinatus, and subscapularis tears were most common in patients with flat acromia (Figure 3A, Table 3) (69.2%, 66.7%, and 56.3%, respectively), followed by curved (23.1%, 25%, and 31.3%, respectively) (Figure 3B) and convex shapes (3.8%, 8.3%, and 6.3%, respectively) (Figure 3C). In the case of supraspinatus and subscapularis muscle tears, hook-shaped acromia (Figure 3D) were noted at the same frequency as convex-shaped (3.8%).

**Table 3:** Frequency of Rotator Cuff Tears (RCTs) Related to the Type of Injury

Name of muscle injury * Type of rotator cuff injury Cross tabulation					
			Type of rotator cuff injury		Total
			Complete tear	Partial tear	
Name of muscle injury	Infraspinatus muscle	Count	3	9	12
		% within name of muscle injury	25.0%	75.0%	100.0%
		% within type of rotator cuff injury	13.6%	10.7%	11.3%
		% of total	2.8%	8.5%	11.3%
	Subscapularis muscle	Count	3	13	16
		% within name of muscle injury	18.8%	81.3%	100.0%
		% within type of rotator cuff injury	13.6%	15.5%	15.1%
		% of total	2.8%	12.3%	15.1%
	Supraspinatus muscle	Count	16	62	78
		% within name of muscle injury	20.5%	79.5%	100.0%
		% within type of rotator cuff injury	72.7%	73.8%	73.6%
		% of total	15.1%	58.5%	73.6%
Total		Count	22	84	106
		% within name of muscle injury	20.8%	79.2%	100.0%
		% within type of rotator cuff injury	100.0%	100.0%	100.0%
		% of total	20.8%	79.2%	100.0%

Partial RCTs were identified in 84 patients (Table 4, Figures 3B, 3C). Partial tears were most commonly observed to affect the supraspinatus muscle (73.8%), followed by the subscapularis (15.5%) and then the infraspinatus (10.7%); the most commonly identified acromial shape in the case of partial tears was flat (69.4%, 61.5%, and 66.7%, respectively), followed by curved (22.6%, 23.1%, and 22.2%, respectively), convex (4.8%, 7.7%, and 11.1%, respectively), and hooked (3.2% and 7.7%, respectively) in the case of supraspinatus and subscapularis tears. Hooked acromia were not observed in any cases of partial tears of the infraspinatus muscle (Table 3).

**Table 4:** Distribution of Rotator Cuff Muscle Injuries in Relation to Acromial Shape and Types of Tears

Supraspinatus muscle				Acromial shape				Total
				Convex	Curved	Flat	Hooked	
	Rotator cuff injury	Partial tear	Count	3	14	43	2	62
			% within rotator cuff injury	4.8%	22.6%	69.4%	3.2%	100%
		Complete tear	Count	0	4	11	1	16
			% within rotator cuff injury	0.0%	25.0%	68.8%	6.3%	100%
	Total		Count	3	18	54	3	78
			% within rotator cuff injury	3.8%	23.1%	69.2%	3.8%	100%
	P-value							.642
Infraspinatus muscle								
	Rotator cuff injury	Partial tear	Count	1	2	6	0	9
			% within rotator cuff injury	11.1%	22.2%	66.7%	0	100.0%
		Complete tear	Count	0	1	2	0	3
			% within rotator cuff injury	0.0%	33.3%	66.7%	0	100.0%
	Total		Count	1	3	8	0	12
			% within rotator cuff injury	8.3%	25.0%	66.7%	0	100.0%
			% of total	8.3%	25.0%	66.7%	0	100.0%
	P-value							.712
Subscapularis muscle								
	Rotator cuff injury	Partial tear	Count	1	3	8	1	13
			% within rotator cuff injury	7.7%	23.1%	61.5%	7.7%	100.0%
		Complete tear	Count	0	2	1	0	3
			% within rotator cuff injury	0.0%	66.7%	33.3%	0.0%	100.0%
	Total		Count	1	5	9	1	16
			% within rotator cuff injury	6.3%	31.3%	56.3%	6.3%	100.0%
			% of total	6.3%	31.3%	56.3%	6.3%	100.0%
	P-value							.487

Complete RCTs are illustrated in Figure 3A/D, with the supraspinatus being the most commonly affected muscle (72.7%). The flat acromial shape was the most frequently identified in the case of complete RCTs (68.8%), followed by curved (25%) and hooked (6.3%) (Table 3). A convex acromion was not observed in combination with a complete RCT.

Among both male and female patients, partial tears (79.5%) were more common than complete RCTs (20.5%) on both the right and left sides. The predominant acromial shape among male patients with partial right-sided RCTs was flat in eight cases, while flat and curved were equally prevalent among male patients with partial left-sided RCTs in five cases. The predominant acromial shape among female patients with partial or complete RCTs of either side was flat (right side = 24 cases and left = 21 cases). In women with left-sided RCTs, partial tears (24 cases) were more common than complete tears (five cases), and the predominant acromial shape was flat (complete tears = 5 cases; partial tears = 16 cases) (Figures 2, 3A).

We found no significant association between sex and the affected shoulder side, which has not been investigated in any recent studies (p = 0.709).

Finally, there was no significant correlation between sex and the acromial shape (p = 0.104); however, we found that the most common acromial shape in patients who had RCTs was flat, both in men (53.3%) and women (76.3%).

## Discussion

Rotator cuff injuries can be assessed using several imaging modalities. In the present study, we used shoulder MRI to evaluate the relationship between the occurrence of partial or complete RCTs and acromial shape, as multiple studies have proven this modality to be the most useful diagnostic test for rotator cuff injury [4, 12-13, 16].

We found no significant association between sex and the affected shoulder side, which has not been investigated in any recent studies. However, our finding of a lack of a significant correlation between these factors (p = 0.709) supports the results of a previous study [11].

We found a flat acromion to be the most prevalent type among all patients with RCTs. Although in line with the results of an earlier study, we did not find a significant relationship between acromial shape and sex [12]. By contrast, a study conducted by Paraskevas et al. reported the flat acromial shape to be strongly associated with female sex [14]. In contrast, the hooked shape was found to be significantly more common among male patients. We believe that the reason for the discrepancies between these studies may be related to genetic and osteological factors.

We identified a significant correlation between supraspinatus injury, acromial shape, and sex (p = 0.030). However, for infraspinatus and subscapularis muscles, we did not identify any significant correlation between acromial shape, muscle injury, and sex. The study by Balke et al. supports our results, reporting that there is no significant correlation between acromial shape, RCT (either partial or complete), and sex [3]. In fact, a study involving 100 patients reported there to be no statistically significant relationship between acromial shape and RCT [15]. By contrast, Balke et al. reported a significant correlation between hooked acromial shape and the occurrence of RCTs [3]. This is corroborated by a study that reported the hooked acromial shape to be the only shape showing a significant relationship with RCTs [11]. However, it was contradicted by another study that reported no correlation between acromial shape even hooked and the occurrence of RCTs [16]. A systematic review and meta-analysis by Morelli et al. reported that patients with a Type III (hooked) acromion are three times more likely to experience RCTs than patients with Type I or Type II acromia [17].

Limitations

The present study has some limitations that should be acknowledged. First, rotator cuff injuries may be influenced by factors, such as degenerative or inflammatory shoulder disease, which were the exclusion criteria in this study. Second, the sample size was small, and patients were recruited from a single center. Therefore, future multicenter studies involving more patients are required to completely confirm the association of acromial shape and RCTs.

## Conclusions

In summary, in the context of RCTs, there is no correlation between the four acromial shapes and sex, regardless of the muscle that is injured. However, we identified a significantly higher incidence of partial supraspinatus tears in the right shoulders of women aged ≥ 50 years with flat acromia. The relationship of sex with other RCTs does not appear to be significant.
